# Content validity of the PROMIS® pediatric family relationships measure for children with chronic illness

**DOI:** 10.1186/s12955-018-1030-8

**Published:** 2018-10-19

**Authors:** Kathryn E. Flynn, Harald Kliems, Nikita Saoji, Jacob Svenson, Elizabeth D. Cox

**Affiliations:** 1 Department of Medicine, Medical College of Wisconsin, 9200 W. Wisconsin Ave, Milwaukee, WI 53226 USA; 2 Department of Pediatrics, University of Wisconsin School of Medicine and Public Health, 600 Highland Ave, Madison, WI 53792 USA

**Keywords:** Qualitative, Content validity, Pediatric, Quality of life, Family relationships, Family interactions

## Abstract

**Background:**

Families play a critical role in supporting the health and well-being of children with chronic illnesses, who face a lifetime of responsibility for self-management of their condition. Our goal was to investigate whether the novel Patient-Reported Outcomes Measurement Information System® (PROMIS®) Pediatric Family Relationships measure, developed primarily within the general pediatric population, reflects the experiences of family relationships for chronically ill children and their parents.

**Methods:**

We conducted semi-structured qualitative interviews with children (aged 8–17) with common chronic conditions: asthma (*n* = 6), type 1 diabetes (*n* = 5), or sickle cell disease (*n* = 5), and separately with one of their parents (*n* = 16). Interviews were recorded, and two team members independently coded the written transcripts facilitated by Nvivo 10. The systematic content analysis used a combination of: 1) pre-specified themes corresponding to the six facets of the domain identified during measure development and reflected in the content of the items (i.e., Sense of Family; Love and Caring; Value and Acceptance; Trust, Dependability, and Support; Communication; Enjoyment), as well as 2) open-coding, allowing participants to define important concepts (i.e., disease impact).

**Results:**

Family relationships were conceptualized in a similar way to the general population, as evidenced by child and parent responses to open-ended questions about family relationships and to specific probes that corresponded with the item content in the Family Relationship 8-item short form. Children spontaneously discussed the impact of their disease on family relationships less often than parents did. Although participants described how living with a chronic illness positively and negatively impacted aspects of family relationships, nearly all participants believed their responses to the PROMIS® Family Relationships items would not change if they (or their child) did not have a chronic illness.

**Conclusions:**

Among a sample of families of children with one of 3 chronic illnesses, participants described family relationships in a way that was consistent with the facets of the PROMIS® Family Relationship domain. This study adds to the content validity of the measure for children with chronic illness.

## Background

Families play a critical role in supporting the health and well-being of children with chronic illnesses, who face a lifetime of responsibility for self-management of their condition. Research in several pediatric chronic conditions suggests that family support can have powerful positive effects on disease self-management and emotional well-being, ultimately resulting in better outcomes [1–8]. However, normal developmental processes, such as separating oneself as an individual, can create challenges in parent-child relationships and family functioning [9]. In fact, children and their parents can have discordant views about their interactions [10, 11], the illness experience [12], and responsibilities for disease self-management [12–14]. To optimize outcomes, families of children with chronic illness must balance the child’s autonomy and ongoing development with behaviors that promote disease management [15–20].

While families play an important role in children’s well-being, few tools systematically assess family relationships from a child’s own perspective. In recognition of this, the National Institutes of Health (NIH) Patient-Reported Outcomes Measurement Information System® (PROMIS®) supported the development of an item bank describing children’s experience with their families, the PROMIS® Pediatric Family Relationships measure. It is intended for children aged 8–17 years (self-report) and parents of children aged 5–17 years (parent report). The PROMIS® standards for measure development and validation include literature review, qualitative and quantitative data collection, and psychometric evaluation using both classical test theory and modern item response theory approaches [21–23]. As such, development of the PROMIS® Family Relationships measure included literature and extant measure review, formative qualitative interviews for concept elicitation with 10 experts as well as with 8 parents and 24 children. Of these interviewees, 3 of the parents had a child with a chronic illness and 8 of the children had a chronic illness. Items were developed, and candidate items then underwent cognitive interviews to evaluate item wording, followed by translatability review and a reading level analysis. Items were psychometrically evaluated in two large field studies [24]. The first of these field studies recruited through schools, primary and specialty care clinics, and an opt-in online panel (Op4G); 146/1845 (18%) of the child participants self-reported a chronic condition. The second field study recruited through a randomly sampled online panel (GfK KnowledgePanel); the presence of chronic conditions in this sample was not assessed. Thus, evidence is needed regarding whether this new measure reflects the experiences of family relationships for chronically ill children and their parents.

The current study is part of a larger, mixed-methods project to advance the understanding of PROMIS® pediatric measures in children with chronic illness, specifically asthma, type 1 diabetes, and sickle cell disease. Over 9 million US children have been diagnosed with one of these conditions, comprising approximately 1/3 of all US children with a chronic illness.

Our goal was to investigate whether the novel Family Relationships measure, developed primarily within the general pediatric population, reflects the experiences of family relationships for chronically ill children and their parents. If it does, the evidence base for the validity of the measure in these populations is increased. To evaluate this, we conducted semi-structured qualitative interviews with children with asthma, type 1 diabetes, or sickle cell disease, and separately with one of their parents. Our research questions were: 1) Among children with asthma, type 1 diabetes, or sickle cell disease and their parents, are family relationships conceptualized in a similar way to the general population? and 2) How does having a chronic illness impact the experience of family relationships for these children and their parents?

## Methods

Children and their parents were recruited by email and phone from existing research registries and in-person at subspecialty clinics at 2 large Wisconsin healthcare systems between March 2016 and April 2017. Interviews were conducted in private rooms on the healthcare campuses. Children and their parents were interviewed separately; on rare occasion, other (young) children were present in the room during parent interviews. Participants had to be a child 8–17 years of age with a provider-confirmed diagnosis of sickle cell disease, asthma, or type 1 diabetes or their parent, and able to speak and understand English. Using maximal variance sampling [25], we targeted a diverse sample of participants on key variables: child age, sex, race/ethnicity, and diagnosis. We monitored recruitment continuously to ensure diversity of the sample across these characteristics (Table 1). All participants provided written informed consent (parents) and/or assent (children); during the consent process, participants were informed about the researchers’ rationale for the study. We offered participants $50 as compensation for their time. The study was approved by the Children’s Hospital of Wisconsin Institutional Review Board.

**Table 1:** Characteristics of child and parent participants^a^ (*N* = 30)

Characteristic	Child % (n)	Parent % (n)
Child’s chronic condition		
Asthma	33% (5)	33% (5)
Type 1 Diabetes	33% (5)	33% (5)
Sickle Cell Disease	33% (5)	33% (5)
Age of child		
8–11	40% (6)	
12–14	33% (5)	
15–18	27% (4)	
Sex		
Male	53% (8)	33% (5)
Female	47% (7)	67% (10)
Race		
White	20% (3)	20% (3)
Black or African American	73% (11)	73% (11)
Other	0% (0)	0% (0)
Ethnicity		
Not Hispanic or Latino	87% (13)	93% (14)
Hispanic or Latino	7% (1)	0% (0)

^a^Values may not add to 100% due to rounding or non-response

Trained interviewers conducted the in-person interviews following a semi-structured interview guide. The interviewers were not involved in formulating the research questions and had no relationship with the interviewees outside of the interview. The guide was pilot-tested with three children and three adults. It began with broad, open-ended questions about family composition, interests, and general family life. Because this work was undertaken to assess evidence for the validity of the PROMIS® measure, the guide also included specific questions corresponding to the content of the 8-item Family Relationships short form. Participants were then asked to complete the 8-item short form. Subsequently, they were probed about the acceptability and utility of answering questions related to family relationships during healthcare visits, the results of which will be reported separately. Finally, the interviewer asked questions about how having a chronic illness did or did not affect family relationships and whether interviewees thought their answers to the 8-item short form would be different if they (or their child) did not have a chronic condition. Interviews were audio-recorded and fully transcribed. The median length of interviews for parents (28 min) was slightly longer than for children (20 min). Interviewers made field notes immediately after completing each interview. These notes recorded information about the interview that may influence the interpretation and coding of the written transcript. The notes were shared with the study team and available to the coders.

### Data analysis

Written transcripts were managed with the qualitative data analysis software NVivo 10. Our data collection and analysis were conducted through an iterative process with each informing the other. The research team had regular discussions in order to: 1) refine the interview guide as needed, 2) develop and refine a codebook, and 3) assess whether new themes were emerging as interviews were coded, which informed our decision to not seek additional interviews (i.e., data saturation) [26, 27].

We conducted a systematic content analysis of each transcript using a combination of: 1) directed content analysis for pre-specified themes corresponding to the six facets of the domain identified during measure development and reflected in the content of the items, as well as 2) open coding, allowing participants to define important concepts [28]. These simultaneous approaches addressed our 2 distinct research questions [29, 30]. We coded participant responses based on their content, that is, regardless of the question that preceded it. Our team-based coding process was guided by a formal codebook that included a definition of each code with separate inclusion/exclusion criteria to clarify how codes differed from each other and should be applied [29]. Two trained coders independently coded all of the data, meeting regularly with each other and the larger research team to ensure consistent application of the codes and to resolve coding discrepancies through discussion, an important component to support reliability of the coding process [26]. To assess whether chronically ill children and their parents conceptualize family relationships in a manner similar to the general population of children and parents, we describe the themes found in the interviews and report the occurrence of each theme across interviews categorized by role (child/parent) and disease type. To understand whether having a chronic illness (or having a child with a chronic illness) impacts family relationships, we describe how children and parents discussed disease impact as well as an analyses of when this theme appeared during the interviews, that is, whether spontaneously or in response to probes from the interviewer.

## Results

### Sample characteristics

Following purposive sampling techniques, interviewees from all three chronic conditions were evenly represented and child participants across the age spectrum between 8 and 17 years old were included (Table 1). The majority of interviewees were Black/African American, consistent with the demographics of children with asthma and sickle cell disease. The sex of participants was evenly distributed among the child participants, but 2/3 of participating parents were women.

### Themes

Our final set of codes comprised the themes described by the six facets of the PROMIS® Family Relationships domain plus additional themes of family composition and the health and disease experience (Table 2). Initially we included a separate code for “Autonomy,” which ultimately was used infrequently and therefore subsumed into the Trust, Dependability, and Support code in the final codebook. With few exceptions, all codes were present in all interviews, that is, there were no clear patterns in presence of themes by role or disease type (Table 3).

**Table 2:** Coding scheme including major themes and their definitions

Theme	Definition
Family Composition	Naming the people considered members of one’s family, without limitations as to living situation.
Facets of Domain	
*Sense of Family*	The consistency of the child’s experiences of being an integral part of the family based on feelings of closeness and connection with parents and family members.
*Trust, Dependability, and Support*	The consistency of the child’s experiences that parents and family members will ‘be there’ to provide help and guidance when they are needed; that family members can be trusted and can be depended on.
*Love and Caring*	The consistency of the child’s sense of being cared about and loved by parents and other family members; having and sharing feelings of affection, warmth, and fondness.
*Value and Acceptance*	The consistency of the child’s feelings of being important in the family, that he/she matters to other family members; child’s experience that parents and other family members understand him/her and approve of him/her.
*Enjoyment*	The consistency of receiving pleasure and having fun in interactions with parents and family members.
*Communication*	The consistency with which parents and family members are available and willing to listen and talk, and child feels understood in interactions with them.
Disease Impact	Responses that reference the experience of having a chronic disease and its impact on one’s life and family relationships.

**Table 3:** Prevalence of themes during interviews, overall and by role and child’s chronic condition

Themes	# of interviews containing theme (*n* = 32)	Child			Parent		
		Asthma (*n* = 6)	Type 1 diabetes (*n* = 5)	Sickle cell disease (*n* = 5)	Asthma (*n* = 6)	Type 1 diabetes (*n* = 5)	Sickle cell disease (*n* = 5)
Family Composition	31	6	5	5	5	5	5
Facets of Domain							
Sense of Family	29	6	4	3	6	5	5
Support/Trust and Dependability	30	5	5	5	5	5	5
Love and Caring	25	4	3	3	5	5	5
Value and Acceptance	31	6	5	5	5	5	5
Enjoyment	31	6	5	4	6	5	5
Communication	28	5	4	4	5	5	5
Disease Impact	30	5	5	5	6	5	4
Pre-probe^a^	16	1	3	1	3	4	4
Post-probe^a^	29	5	5	5	5	5	4

^a^In interview guide, probes about disease impact were placed at the end of the interview, so in coding we distinguished between when these themes were found pre- and post-probing

### Conceptualizations of family relationships

All parent and child responses to the open-ended question about what makes family relationships strong were found to align with one or more of the six facets of the PROMIS® family relationships measure (defined in Table 2). Parents described close family relationships as requiring communication, time together, and supporting, caring for, and/or valuing the other members. For example, the father of 13-year-old said, “Communication, trust, honesty, just time, you know, being spent with each other … well I guess being there for each other.” Similarly, the mother of 8-year-old said, “Just communicating, just being together […] being honest with one another” and the mother of 12-year-old said, “Communication makes our relationship strong so it’s, you know, talking all the time, asking lots of questions, and listening to each other.” Children included these and other concepts like love in their descriptions. For example, a 12-year-old boy said, “To, like, be able to talk to them, know that they love you, and to basically know that they’re always there for you,” while a 13-year-old said, “It’s a lot of love in [my] family.” An 11-year-old girl said, “I think it’s like being able to talk to them whenever you need to, tell them secrets without them telling anybody.” A 15-year-old girl said, “You gotta have a lot of trust and loyalty;” a 13-year-old girl said, “It’s like you have someone that’s always there for you,” and a 14-year-old boy said, “Like they’re always communicating.” Finally, another 12-year-old boy included the concept of spending time together, “When they ask, like, to talk to you, or when they ask if you want to go somewhere like to the mall, for example, or if you, like, just want to hang out.”

The *Family Composition* theme covered how participants defined their families, regardless of whether they lived in the same household or not. While participants had different criteria for defining their families, nobody had difficulty naming who they considered their family. For example, a 16-year-old girl who lived only with her mother described her family as “the everyday people” who included her mom, step-siblings, and an uncle, all of whom she spoke about regularly throughout the interview.

*Sense of Family* covered feelings of belonging, closeness, and connection to family members as well as the strength of family relationships. A 13-year-old girl described the connection with her father, “Like, just us in the car, it’s like, he’ll like bring up a subject and I’ll like, laugh at him then I’ll make a comment. It’s like a father-daughter conversation.” The mother of an 8-year-old boy described strong family relationships as, “Just having family time together. I’m learning it now, you know. Putting away the technology stuff and everything, just—just being together as a family, even if it’s like Sunday dinners coming together or game night or something like that.” Belonging was expressed by the father of a 13-year-old girl as, “I’ve been there for everything. [Child’s name] knows she’s just – that’s – that’s like my heart. That’s my daughter, that’s my princess, so I don’t know. I can’t see her even thinking that she just belong anywhere else.”

*Trust, Dependability, and Support* covered concepts of helping, providing guidance, and being there when needed. For example, a 12-year-old boy said, “To basically know that they’re always there for you,” and the mother of a 14-year-old boy described, “How everybody’s willing to help out each other. When there’s a problem, they’re there, and when there’s something good, they’re there to celebrate. So they’re always there.” Within this code we included the concepts of being treated fairly (or unfairly) as well as comparisons to others provided as a metric for fairness. For example, a 16-year-old boy described feeling like he was treated differently from his older brother: “I see my brother like all the time, like he just goes out on the weekends and stuff and just hangs out, but it’s probably ‘cause he can drive and stuff, but like with me like it’s almost like they know, like want to know, everything I’m doing at every moment of the day.” A 13-year-old boy described receiving help from his mother, “About struggling in school, just giving me advice like how to stay positive and like cause at the time I was struggling I had my grades low, she was like just stay positive. Give me good advice and like let the negativity flow away.”

Another aspect of the Trust, Dependability, and Support theme was the concept of developing independence and increasing responsibilities for disease self-management. For example, the mother of a 10-year-old described how her son “tries to do everything by himself. He don’t want to feel like he can’t do anything, so it depends on him pretty much. We help him a lot during his down days, but if he’s not feeling too bad, he does a lot by himself.” Two of the older children and a few parents also made comments that addressed children’s autonomy and/or independence. For example, a 16-year-old girl spoke about how having diabetes requires her to be responsible and how she struggled with finding the right balance of independence with still managing her chronic condition adequately. She talked about turning down offers of help from her mother and others, even when that meant she could not live up to the responsibility of managing her condition, which sometimes resulted in negative outcomes, saying “I be more mad at myself ‘cause it’s like I failed myself but I do it by myself, so it’s like sad ‘cause I’m only 16 and having to do all this; like diabetes not easy. I got a disease, an illness where I do need to take better care of it […] but then it’s like now that I’m so used to doing it by myself, I don’t want nobody to help me with it. Like when my mom would try to help me and I be like, she’ll be like, ‘what was your blood sugar was?’ and that irritates me ‘cause I’m like: Why? Why do you care?” The mother of a 14-year-old discussed how giving too much attention could be detrimental to children needing “to be able to branch out and do things on their own. So if you’re doing things for them or giving them too much attention while they’re trying to do stuff, then that’s too much.”

*Love and Caring* covered the sense of being cared about and loved by family members. A 10-year-old noted “a lot of hugs and kisses” as a mark that her family loved each other, and others also mentioned physical affection or saying “I love you” as a sign of love and caring. The father of 16-year-old girl said, “If you don’t feel loved or don’t feel wanted, you know, that’s kind of the base building block of things. You know, of human – human wants.” The mother of 14-year-old boy described caring for her son by encouraging him, “Well, we always try to tell him no matter what you do, put your best foot forward. And even if you don’t do as well as everybody else, you do your best and you’re great.” She also described the lack of love her son feels because his father is absent, “His dad’s nowhere around and he hates it because he doesn’t understand […] why his dad doesn’t love him. So I have to make him understand that it’s his dad’s loss, it’s not his loss.” This theme also included family members paying attention to each other. The father of a 16-year-old boy described, “We tell him we love him, he knows we’re engaged, he knows we’re involved with his day-to-day life and his success and what he wants to do and how he needs to do it.”

*Value and Acceptance* covered feeling important (or conversely feeling like a burden), feeling like one matters to their family members and that family members make them feel good and understand them. A 16-year-old girl described the importance of her “leadership skills” to her family, because “They feel comfortable leaving me with my younger siblings and kind of like taking charge of them when I need to.” Parents described making sure their children are noticed and appreciated, giving examples of good grades in school, participation in activities, completing chores at home, etc. The mother of 8-year-old boy said, “I think that’s very important to feel, you know, like somebody, like you’re worthy to be loved or liked or hugged or being around.” The mother of 13-year-old described how she demonstrated value and acceptance to her son, “We always acknowledge the good work that he does. We acknowledge the bad things that he does too […] I’m a believer in rewards and consequences, because it’s just life.” One child, a 16-year-old girl, described feeling like a burden to her family and not feeling important to them, “When I be in hospitals and I be in there by myself [….] That be the depressing moment I have because then they got to have me on suicide watch because I’m by myself and my thoughts is getting to me and I don’t have nobody there […] not even my mom ‘cause don’t nobody want to deal with it.” This was corroborated in the interview with her mother who said, “Right now at this present time, she don’t feel like she’s important to the family […] she’s not feeling too accepted by them at this time.”

*Enjoyment* covered having fun or pleasurable interactions with family members. Many children described holidays, birthdays, and trips out of town as enjoyable times with family. An 11-year-old girl described, “We go to my grandpa’s every Sunday and Wednesdays, so and my cousins come, so it’s pretty fun too.” An 8-year-old boy described his favorite leisure activities as, “Play on the iPad or play with my cousins […] we play games, we play hide and go seek.” One 9-year-old boy said he did not usually have fun with his family because they didn’t do things together, “We just do our own thing […] Like I play my [video] games, my brother watches TV, and they [parents] usually watch TV.” Parents often described eating together, travel, and shared activities such as playing games or sports. The mother of 13-year-old boy indicated that having fun together was an important part of being a family, “Because sometimes families miss out on that. You know, you get so caught up in your own personal life, you miss what it’s all about. ‘Cause see whenever – growing up as a child for me, that’s all I had was cousins and family. I didn’t really have outside friends. All I had was family and that’s all we did was together every day all day, that’s all we did was play.” Another mother described organizing fun activities for her family because, “It builds the memories and it keeps [us] close.”

*Communication* covered listening, talking and willingness to talk, and the communication outcome of feeling understood. The father of 11-year-old girl said, “We have an open door policy with them, like when they come and they want to talk about stuff, whatever’s on their mind, whatever’s concerning to them, if it’s something that’s going on in school to all the way down to – I would love to get some ice cream. You know what I mean, like it’s – nothing’s off limits to them.” Similarly, the mother of 13-year-old boy said, “You know sometimes you can’t talk, you have to listen. Every time you can’t talk. You just have to be the ears and listen to hear what’s going on. So that’s very important and something you can understand how a person is feeling, where they’re coming from, or what their point is what they’re trying to explain. You just have to listen.” In the interviews with children, many gave general responses indicating that their parents listened “all the time” or “a lot.” Some children provided concrete examples of a parent listening to them, such as the 11-year-old who said, “We’re planning on going vacation on like a huge vacation […], we were like discussing stuff, where we’re gonna go, and what house to choose.”

*Disease Impact* included the experience of living with a chronic illness, including how illness does or doesn’t impact family interactions and relationships. The large majority of both parents and children indicated that they had not thought about their (or their child’s) illness when they answered the general questions about family relationships during the interview. For example, a parent of an 11-year-old who explained, “I very rarely think about her specifically with diabetes. It’s more of ‘she’s my child’ and then that’s it. You know, diabetes just came with it, so it doesn’t define who she is.” However, parents and children may have differing views about the relevance of the child’s chronic disease to the concept of family relationships, as evidenced by whether they spontaneously raised the chronic condition during probing about family relationships. Among the 14 child participants who mentioned their chronic illness, 1 of 4 children with asthma, 3 of 5 children with diabetes, and 1 of 5 children with sickle cell disease spontaneously raised their chronic illness while being probed about family relationships. Parents more commonly brought up their child’s illness without being probed specifically about it. Among parents, 3 of 5 parents of a child with asthma, 4 of 5 parents of a child with diabetes, and 4 of 4 parents of a child with sickle cell disease spontaneously brought up their child’s chronic illness when asked questions about family relationships.

Children across each of the 3 chronic conditions described ways that aspects of their family relationships can be influenced by these conditions, such as being able to have fun or play. Specifically, children with asthma described becoming out of breath when playing or running or because of allergies and needing to carry with them and use an inhaler – something that for some children involved the family helping with those responsibilities. For example, as a 13-year-old described: “Having asthma’s like, it’s like some things I can’t do like when I want to. When I take my inhaler. I go home, my family they make sure I’m alert. They make sure [to] have inhalers with them at all the times. Like in their car or in their purse or something.” A mother of a 14-year-old described how her son’s diagnosis affected their family, “When [child name] was diagnosed with diabetes, everyone was there to help him. We all got together, we all learned how to help him, we all changed our diet to make it easier for him. If he couldn’t have something, nobody got it. We all started learning how to count carbs [….] Makes that transition a lot easier for the one person who has to do it.” A 16-year-old boy with sickle cell disease said, “I think like having sickle cell like, that could be why my family like cares so much around me or like watches like – pays like a lot of attention to me, just like to make sure I’m doing ok and like never am in pain and […] can’t do anything about it.” At least one child described less positive views of the influence of her chronic condition on family relationships. This 16-year-old girl thought having diabetes changed her role in her family, “Yeah, ‘cause I feel like the black sheep or like the burden child.”

## Discussion

The primary purpose of this study was to evaluate the content validity of the PROMIS® pediatric Family Relationships measure among children with chronic conditions and their parents. In 32 qualitative interviews with 16 child–parent dyads, we found that children with chronic conditions and their parents conceptualize family relationships in a manner similar to the general population. This conclusion is supported by responses to the open-ended questions describing what makes family relationships strong, where all responses aligned with one or more of the six facets of the PROMIS Family Relationships domain. Further support for the instrument’s content validity lies in the alignment of participants’ responses to the specific probes that corresponded with the item content in the Family Relationships short form. Our sample was diverse with regard to child age and diagnosis. Few tools systematically assess family relationships from a child’s own perspective, and the PROMIS Family Relationships item bank offers a robust approach to this important domain.

Our second goal was to understand how having a chronic illness affected the experience of family relationships for children and their parents. Most participants said their answers to the PROMIS® Family Relationships short form would not change if they did not have a chronic illness, yet they also articulated how living with a chronic illness impacted various aspects of family relationships. One noteworthy pattern we observed was that the majority of parents spontaneously raised the child’s chronic condition while discussing family relationships, while children did this less frequently. This difference is consistent with previous research that has shown discordant views between children and their parents about their interactions [10, 11] and experience of illness [12].

In our interviews, the concept of autonomy or independence of the child was discussed as part of family relationships. This concept is not directly mentioned in the definitions of the Family Relationships facets nor reflected in the items. In the development of the PROMIS Family Relationships item bank, some items were tested that may be related to this concept, e.g., “My parents told me what they wanted me to do” and “My parents made sure I did what I was supposed to do”. These items were part of a candidate facet called Predictability but were ultimately eliminated from the item bank because of low factor loadings with the rest of the facets of family relationships [24]. However, in addition to developing independence from family and parents in general, children with a chronic illness also need to transition toward more independence in their disease self-management [15–20]. Our results were consistent with this focus on autonomy and responsibility with regard to disease self-management. We coded text reflective of it under the Trust, Dependability, and Support facet, which covers helping, providing guidance, and being there when needed. While the previously-tested items did not fit psychometrically, new items related to developmentally-appropriate independence could be tested in a future version of the PROMIS® Family Relationships item bank.

Our study has several limitations. The geographic diversity is limited to Wisconsin, though we recruited participants from two large centers that capture both rural and urban residents. All participants were either white or African American; we had limited Hispanic representation and no Asian or other races represented. We included only children 8–17 years of age and their parents, aligning with PROMIS®‘s goal of self-report for children 8–17 years of age. Parent reports have been evaluated for children as young as 5 years of age, but we are unable to comment on the validity of these parent reports of family relationships for younger children. Finally, we did not track reasons for non-participation, nor did we return transcripts or study results to participants for their comments or corrections.

## Conclusion

For the PROMIS® Family Relationships measure to achieve wide use, it must be supported by evidence for validity in multiple contexts. This measure underwent a rigorous development process [24], and this study adds to the content validity of the measure for children with chronic illness. Although broadly generalizing our findings to children with other chronic illnesses should be done with caution, the conceptualization of family relationships in these three different chronic conditions appears analogous. In addition, the influences of chronic conditions on aspects of family relationships described by our participants are also likely analogous, such as supporting disease management and developing new skills or family behaviors in response to the condition. These conclusions complement on-going quantitative work demonstrating the reliability and validity of the measure among children with chronic illness and their parents [30]. Use of the PROMIS® Family Relationships measure has the potential to help healthcare organizations and researchers quantify family relationships to identify needs and also to compare the outcomes from existing family-focused interventions for improving pediatric chronic illness care.

## Abbreviations

NIH: National Institutes of Health
PROMIS®: Patient-Reported Outcomes Measurement Information System®

## References

[1] Perez-Marin M, Gomez-Rico I, Montoya-Castilla I (2015). Type 1 diabetes mellitus: psychosocial factors and adjustment of pediatric patient and his/her family. Review. Arch Argent Pediatr.

[2] Moreira H, Frontini R, Bullinger M, Canavarro MC (2013). Caring for a child with type 1 diabetes: links between family cohesion, perceived impact, and parental adjustment. J Fam Psychol.

[3] Raphael JL, Butler AM, Rattler TL, Kowalkowski MA, Mueller BU, Giordano TP (2013). Parental information, motivation, and adherence behaviors among children with sickle cell disease. Pediatr Blood Cancer.

[4] Logan DE, Radcliffe J, Smith-Whitley K (2002). Parent factors and adolescent sickle cell disease: associations with patterns of health service use. J Pediatr Psychol.

[5] Hildenbrand AK, Barakat LP, Alderfer MA, Marsac ML (2015). Coping and coping assistance among children with sickle cell disease and their parents. J Pediatr Hematol Oncol.

[6] La Greca AM, Auslander WF, Greco P, Spetter D, Fisher EB, Santiago JV (1995). I get by with a little help from my family and friends: adolescents’ support for diabetes care. J Pediatr Psychol.

[7] Christiaanse ME, Lavigne JV, Lerner CV (1989). Psychosocial aspects of compliance in children and adolescents with asthma. J Dev Behav Pediatr.

[8] Cutfield SW, Derraik JGB, Reed PW, Hofman PL, Jefferies C, Cutfield WS (2011). Early markers of glycaemic control in children with type 1 diabetes mellitus. PLoS One.

[9] Hazen E, Schlozman S, Beresin E (2008). Adolescent psychological development: a review. Pediatr Rev.

[10] Berg CA, Schindler I, Maharajh S (2008). Adolescents’ and mothers’ perceptions of the cognitive and relational functions of collaboration and adjustment in dealing with type 1 diabetes. J Fam Psychol.

[11] Anderson BJ, Auslander WF, Jung KC, Miller JP, Santiago JV (1990). Assessing family sharing of diabetes responsibilities. J Pediatr Psychol.

[12] Beveridge RM, Berg CA, Wiebe DJ, Palmer DL (2006). Mother and adolescent representations of illness ownership and stressful events surrounding diabetes. J Pediatr Psychol.

[13] Ekim A, Ocakci AF (2013). Perceptions of parents and children regarding asthma management responsibilities. J Spec Pediatr Nurs.

[14] Greenley RN, Josie KL, Drotar D (2006). Perceived involvement in condition management among inner-city youth with asthma and their primary caregivers. J Asthma.

[15] Hanson CL, Henggeler SW, Harris MA, Burghen GA, Moore M (1989). Family system variables and the health status of adolescents with insulin-dependent diabetes mellitus. Health Psychol.

[16] Hauser ST, Jacobson AM, Lavori P, Wolfsdorf JI, Herskowitz RD, Milley JE (1990). Adherence among children and adolescents with insulin-dependent diabetes mellitus over a four-year longitudinal follow-up: II. Immediate and long-term linkages with the family milieu. J Pediatr Psychol.

[17] Hanson CL, La Greca AM, Siegel LJ, Wallander JL, Walker CE (1992). Developing systemic models of the adaptation of youths with diabetes. Advances in pediatric psychology: stress and coping in child health.

[18] Walders N, Drotar D, Kercsmar C (2000). The allocation of family responsibility for asthma management tasks in African-American adolescents. J Asthma..

[19] Helgeson VS, Palladino DK, Reynolds KA, Becker DJ, Escobar O, Siminerio L (2014). Relationships and health among emerging adults with and without type 1 diabetes. Health Psychol.

[20] Rosland AM, Heisler M, Piette JD (2012). The impact of family behaviors and communication patterns on chronic illness outcomes: a systematic review. J Behav Med.

[21] DeWalt DA, Rothrock N, Yount S, Stone AA (2007). Evaluation of item candidates: the PROMIS qualitative item review. Med Care.

[22] Reeve BB, Hays RD, Bjorner JB, Cook KF, Crane PK, Teresi JA (2007). Psychometric evaluation and calibration of health-related quality of life item banks: plans for the patient-reported outcomes measurement information system (PROMIS). Med Care.

[23] PROMIS® instrument development and psychometric evaluation scientific standards. http://www.healthmeasures.net/images/PROMIS/PROMISStandards_Vers2.0_Final.pdf. Accessed 10 Oct 2018.

[24] Bevans KB, Riley AW, Landgraf JM, Carle AC, Teneralli RE, Fiese BH (2017). Children’s family experiences: development of the PROMIS® pediatric family relationships measures. Qual Life Res.

[25] Jansen H. The logic of qualitative survey research and its position in the field of social research methods. Forum Qual Soc Res. 2010;11.

[26] Lasch KE, Marquis P, Vigneux M, Abetz L, Arnould B, Bayliss M (2010). PRO development: rigorous qualitative research as the crucial foundation. Qual Life Res.

[27] Saunders B, Sim J, Kingstone T, Baker S, Waterfield J, Bartlam B (2018). Saturation in qualitative research: exploring its conceptualization and operationalization. Qual Quant.

[28] Hsieh HF, Shannon SE (2005). Three approaches to qualitative content analysis. Qual Health Res.

[29] MacQueen K, McLellan E, Kay K, Milstein B (1998). Codebook development for team-based qualitative analysis. Cultural Anthropol Methods.

[30] Cox ED, Connolly JR, Palta M, Rajamanickam VP, Flynn KE. Reliability and validity of PROMIS® Pediatric Family Relationships Short Form in children with chronic disease. Qual Life Res. 2018;27(Suppl 1):S53–S54. 10.1007/s11136-019-02266-x PMC6980213 31401748

